# Accurate Mass Identification of an Interfering Water Adduct and Strategies in Development and Validation of an LC-MS/MS Method for Quantification of MPI8, a Potent SARS-CoV-2 Main Protease Inhibitor, in Rat Plasma in Pharmacokinetic Studies

**DOI:** 10.3390/ph15060676

**Published:** 2022-05-27

**Authors:** Yang Wang, Huan Xie, Yugendar R. Alugubelli, Yuying Ma, Shiqing Xu, Jing Ma, Wenshe R. Liu, Dong Liang

**Affiliations:** 1 Department of Pharmaceutical Sciences, College of Pharmacy and Health Sciences, Texas Southern University, Houston, TX 77004, USA; yang.wang@tsu.edu (Y.W.); huan.xie@tsu.edu (H.X.); jing.ma@tsu.edu (J.M.); 2 Department of Chemistry, Texas A&M University, College Station, TX 77843, USA; namaswi@tamu.edu (Y.R.A.); yuyingma.cn@gmail.com (Y.M.); shiqing.xu@tamu.edu (S.X.)

**Keywords:** MPI8, MPI8•H_2_O adduct, SARS-CoV-2, LC-MS/MS, method development and validation, pharmacokinetics

## Abstract

MPI8, a peptidyl aldehyde, is a potent antiviral agent against coronavirus. Due to unique tri-peptide bonds and the formyl functional group, the bioassay of MPI8 in plasma was challenged by a strong interference from water MPI8. Using QTOF LC-MS/MS, we identified MPI8•H_2_O as the major interference form that co-existed with MPI8 in aqueous and biological media. To avoid the resolution of MPI8 and MPI8•H_2_O observed on reverse phase columns, we found that a Kinetex hydrophilic interaction liquid chromatography (HILIC) column provided co-elution of both MPI8 and MPI8•H_2_O with a good single chromatographic peak and column retention of MPI8 which is suitable for quantification. Thus, a sensitive, specific, and reproducible LC-MS/MS method for the quantification of MPI8 in rat plasma was developed and validated using a triple QUAD LC-MS/MS. The chromatographic separation was achieved on a Kinetex HILIC column with a flow rate of 0.4 mL/min under gradient elution. The calibration curves were linear (r^2^ > 0.99) over MPI8 concentrations from 0.5–500 ng/mL. The accuracy and precision are within acceptable guidance levels. The mean matrix effect and recovery were 139% and 73%, respectively. No significant degradation of MPI8 occurred under the experimental conditions. The method was successfully applied to a pharmacokinetic study of MPI8 after administration of MPI8 sulfonate in rats.

## 1. Introduction

SARS-CoV-2 is the coronavirus pathogen of COVID-19, which has been dominating the pandemic since the beginning of 2020. SARS-CoV-2 uses its membrane spike protein to recognize the human receptor ACE2 for infection [1,2]. Luckily, several types of vaccines, especially the mRNA-based vaccines, have been introduced worldwide and show good effectiveness via targeting the spike protein. On the other hand, the virus is continuously evolving and its mutant virus strains could evade the current vaccine protection. The most significant cases were the emergence of the Delta variant in mid-2021 and the vast infection surge from the Omicron variant since December 2021 [3]. Current data show that most of the marketed vaccines are still effective in minimizing hospitalization and the mortality rate from mutant SAR-CoV-2 viruses, such as the Omicron variant [4]. However, maintaining the effectiveness of vaccines and/or updated versions of vaccines for new SAR-CoV-2 virus strains could be challenging. Meanwhile, the extremely high transmissibility and severity of this virus in vulnerable populations are still having a significant impact on everyone around the world. Thus, there is an urgent need to develop effective therapeutics against the virus.

Since November 2020, the Food and Drug Administration (FDA) has granted Emergency Use Authorization (EUA) for multiple monoclonal antibody (mAb) therapies for the treatment of mild-to-moderate COVID-19 in non-hospitalized persons at high risk of severe disease [5], but for non-hospitalized patients, all monoclonal antibodies must be administered by intravenous (IV) infusion at an infusion site [6]. In addition, an effective monoclonal antibody directed only to a specified variant would be utterly ineffective vis-à-vis a newly spread variant of the SAR-CoV-2 virus [7]. 

Oral antiviral agents are a significant step in expanding the toolbox of treatments outside of a hospital setting. In December 2021, the FDA issued EUAs for two small-molecule oral antiviral agents, molnupiravir by Merck Co. and nirmatrelvir-ritonavir (Paxlovid) by Pfizer, for the outpatient treatment of mild to moderate COVID-19 patients who are at risk of progression [8]. Both treatments can be taken orally at home and have been shown to reduce the likelihood of a person needing to receive hospital care for a COVID-19 infection. Molnupiravir is a prodrug that is converted to form the active ribonucleoside triphosphate (NHC-TP) in vivo. NHC-TP inhibits viral replication via incorporation into the SARS-CoV-2 RNA genome. Nirmatrelvir is a potent SARS-CoV-2 main protease (M^Pro^) inhibitor. Boosting nirmatrelvir with ritonavir, a potent CYP3A inhibitor, leads to increased plasma concentrations of nirmatrelvir for better antiviral efficacy [9,10].

**Figure 1:**
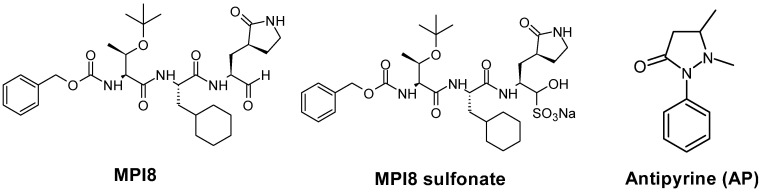
Chemical structures of MPI8, its prodrug MPI8 sulfonate, and antipyrine (AP, internal standard).

As an essential enzyme, M^Pro^ is an established target for the development of SARS-CoV-2 antivirals, as exemplified by Paxlovid [11,12,13]. Another major target is cathepsin L, a cysteine protease, that is a known host protease playing a vital role in SARS-COV-2 replication and pathogenesis [14]. In previous studies, we developed a series of M^Pro^ inhibitors, MPI1-10, and characterized both their M^Pro^ inhibition potency and their antiviral potency [15,16]. As a tripeptidyl aldehyde (Figure 1), MPI8 shows the highest antiviral potency, with an EC_50_ value of 30 nM, to neutralize SARS-CoV-2 (USA-WA1/2020) in Vero E6 cells [17]. In addition, MPI8 is potent toward cathepsin L, but not toward any other tested proteases that can exert dual functions, with high selectivity in inhibiting both cellular M^Pro^ and cathepsin L over cathepsins B and K. Due to its high selectivity toward cathepsin L that reduces potential toxicity toward host cells and high cellular and antiviral potency, MPI8 is a promising candidate for preclinical and clinical investigations for treating COVID-19. To facilitate the preclinical drug development process, we developed a sensitive, specific, and reproducible LC-MS/MS method for the quantitation of MPI8 in rat plasma samples.

MPI8 is provided as MPI8 sulfonate (an aldehyde bisulfite), a prodrug that possesses physicochemical characteristics of an oral drug candidate (Figure 1). This prodrug strategy has been used in GC376, a similar aldehyde bisulfite that can quickly convert into its active aldehyde form [18,19]. Just like GC376, MPI8 sulfonate rapidly converts to MPI8 in vivo. MPI8 is a tripeptidyl aldehyde warhead, making it an ideal candidate to form an adduct and/or hydrate with solvents, such as water and methanol. This could be a very challenging issue in terms of developing an LC-MS/MS bioanalytical assay because it would often not differentiate a parent compound from its co-existing forms due to the possibility of in-source fragmentation and the formation of ion adducts in mass spectrometry. Such compounds are usually in equilibrium during bio-sample analysis until after HPLC column separation. Here, we present an LC-MS/MS quantification of MPI8 in a biospecimen with special highlights in the identification of an MPI8 water adduct and suitable column selections to develop a reliable assay method.

## 2. Results and Discussion

### 2.1. Identification of MPI8 Water Adduct (MPI8•H_2_O) in Rat Plasma

**Figure 2:**
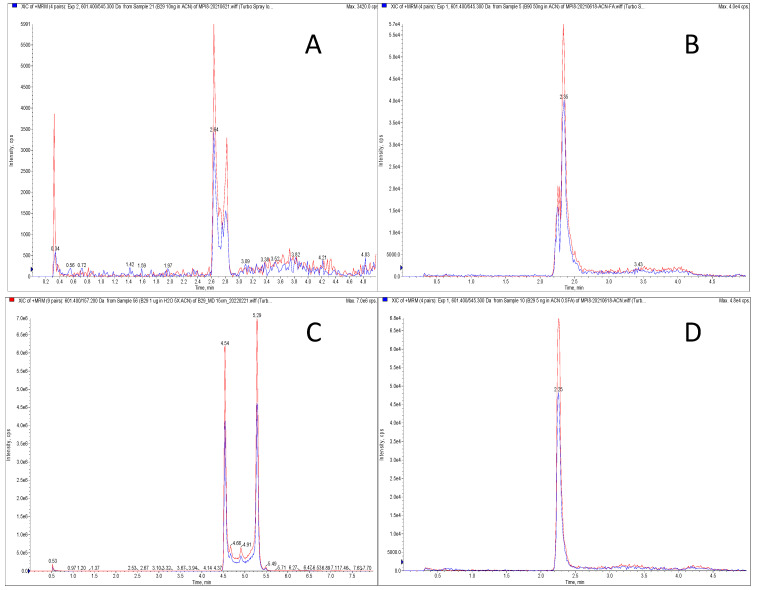
MRM chromatograms for MPI8 (transitions *m*/*z* 601.4→545.3 and 601.4→157.2): Not-fully-separated multiple peaks on a Kinetex F5 column (**A**) and an Acquity HSS-T3 column (**B**); distinctly separated double peak chromatograms of MPI8 on a Synergi Fusion-RP column (**C**); single peak with good peak shape on a Kinetex HILIC column (**D**). The time program of the gradient: (**A**,**B**,**D**) Phase B was initially kept at 5% for 0.5 min, increased from 5% to 90% in the next 2.5 min, then decreased to 5% in 1.0 min, and kept stably at 5% for 1 min; (**C**) Phase B was initially at 20% for 0.5 min, increased from 20% to 90% in 4.5 min, kept at 90% for 2 min, decreased to initial concentration (20%) in 1 min and equilibrated for 2 min.

**Figure 3:**
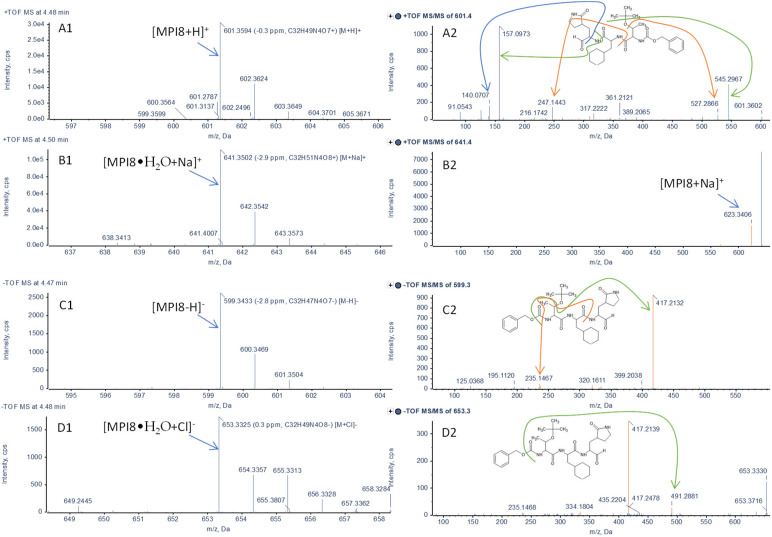
HR MS and MS/MS spectra of MPI8 and MPI8•H_2_O using an extracted sample from MPI8 spiked plasma at retention time of 4.5 min after column elution on an X500B QTOF mass spectrometer; (**A1**,**A2**) and (**B1**,**B2**) in positive mode, (**C1**,**C2**) and (**D1**,**D2**) in negative mode; the proposed fragmentation of product ions are shown on MS/MS spectra: (**A1**) protonated MPI8 [MPI8 + H]^+^ MS (*m*/*z* 601.3594), (**A2**) protonated MPI8 [MPI8 + H]^+^ MS/MS; (**B1**) [MPI8•H_2_O + Na]^+^ MS (*m*/*z* 641.3502) (**B2**) [MPI8•H_2_O + Na]^+^ MS/MS; (**C1**) deprotonated MPI8 [MPI8 −H]^−^ MS (*m*/*z* 599.3433), (**C2**) deprotonated MPI8 [MPI8 − H]^−^ MS/MS; (**D1**) [MPI8•H_2_O + Cl]^−^ MS (*m*/*z* 653.3325), (**D2**) [MPI8•H_2_O + Cl]^−^ MS/MS.

**Figure 4:**
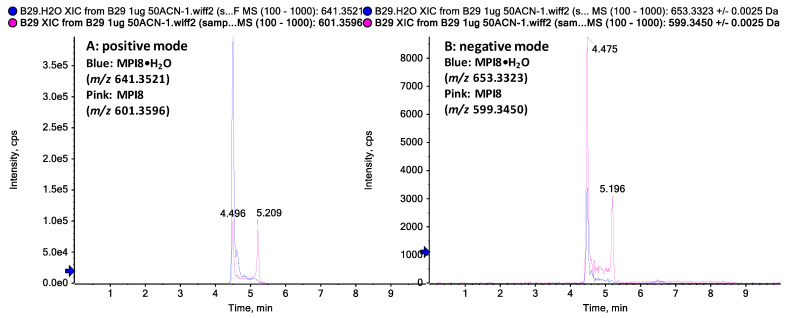
HR-MS extracted ion chromatograms of MPI8 and MPI8•H_2_O eluted on a Synergi Fusion-RP column; the peak at 5.2 min was MPI8; the peak at 4.5 min was MPI8•H_2_O, and the MPI8 signal was due to the in-source fragmentation: (**A**) MPI8 ([MPI8 + H]^+^) *m*/*z* 601.3596 in pink and MPI8•H_2_O ([MPI8•H_2_O + Na]^+^) *m*/*z* 641.3521 in blue in positive mode (**B**) MPI8 ([MPI8 −H]^−^) *m*/*z* 599.3450 in pink and MPI8•H_2_O ([MPI8•H_2_O + Cl]^−^) *m*/*z* 653.3323 in blue in negative mode.

When MPI8 detection was performed in MRM mode, we monitored two transitions of protonated MPI8 ([MPI8 + H]^+^) to two respective fragments, i.e., *m*/*z* 601.4→545.3 and 601.4→157.2 (Table 1). Under both transition windows, MPI8 showed two separate peaks with high baseline interference between the peaks. This phenomenon was apparent in several generally used reverse-phase (RP) columns (Phenomenex Synergi Fusion-RP, Waters Acquity HSS-T3, Phenomenex Kinetex F5, Figure 2). Not-fully-separated multiple peaks were shown on a Kinetex F5 (Figure 2A) and an Acquity HSS-T3 column (Figure 2B), and distinctly separated double peak chromatograms of MPI8 were shown on a Synergi Fusion-RP column (Figure 2C). Furthermore, the chromatogram pattern was consistent, regardless of the sample matrix, i.e., water, acetonitrile, or plasma. We believe that both peaks belong to MPI8 and are not impurity or degradation products. To elucidate the structures of both peaks, we conducted structural identification on an X500B QTOF mass spectrometer using an extracted sample from MPI8 spiked plasma; the LC separation was performed on the Synergi Fusion-RP column under same gradient elution. High-resolution (HR) MS Q1 ions were detected at 4.5 min: *m*/*z* 601.3594 (protonated MPI8, [MPI8 + H]^+^), 623.3414 ([MPI8 + Na]^+^), 641.3502 ([MPI8•H_2_O + Na]^+^), and 657.3255 ([MPI8•H_2_O + K]^+^) in positive mode; and at *m*/*z* 599.3433 (deprotonated MPI8, [MPI8 − H]^−^), 635.3203 ([MPI8 + Cl]^−^), and 653.3325 ([MPI8•H_2_O + Cl]^−^) in negative mode (Figure 3). The data confirmed the co-existence of MPI8 and its water adduct MPI8•H_2_O. Both MPI8 and MPI8•H_2_O were easy-to-form ion adducts, and the protonated/deprotonated ion of MPI8•H_2_O was undetectable, implying the high ionic affinity of MPI8•H_2_O. The HR-MS chromatograms showed MPI8•H_2_O was mostly detected at 4.5 min, and MPI8 was detected at 4.5 and 5.2 min (Figure 4). Thus, we concluded that the peak at 4.5 min was MPI8•H_2_O, and the signals for MPI8 at 4.5 min were due to the in-source fragmentation [20] of MPI8•H_2_O; the peak at 5.2 min was MPI8.

Ion and/or water adducts to an analyte are not uncommon in mass spectrometer detections, especially in the case of electrospray ionization (ESI). ESI is generally viewed as a soft ionization technique, most applicable to polar/highly polar compounds in solution. However, ESI is so ‘‘soft’’ that it can generate not only protonated/deprotonated ions but also different ion adducts ([M + Na]^+^, [M + K]^+^, [M + Cl]^−^, etc.), as well as ion adducts formed with available solvent molecules (H_2_O and CH_3_OH) under different mobile phase conditions [21,22]. However, such adducts often have a limited effect on the quantification of an analyte where the protonated ion [M + H]^+^ (positive mode) or the deprotonated ion [M − H]^−^ (negative mode) are used as the precursor in multiple reaction monitoring (MRM) scan. The existence of these adducts poses a pitfall in overall MS responses to an analyte; thus, efforts have been made to minimize the formation of the adducts during the LC-MS/MS quantification process [23,24]. In some specific cases, the ion and/or water adducts may be used as a precursor for fragmentation when protonated/deprotonated ion of the analyte is least available [25,26,27,28,29]. Water adducts have been carefully discussed when they have shown specialty in structural characteristics or formation mechanisms [22,30,31,32,33]. Therefore, water adducts detected in MS are generally attributed to the in-source formation with solvents. In the present study, the detection of MPI8•H_2_O is not similar to the situation discussed above. Distinct chromatogram peaks of MPI8 and MPI8•H_2_O on a RP column (Figure 4) clearly suggested that MPI8•H_2_O was formed in a sample matrix rather than one generated in an MS detector in the presence of a mobile phase. Further, the observed ion adducts ([MPI8•H_2_O + Na]^+^, [MPI8•H_2_O + K]^+^, and [MPI8•H_2_O + Cl]^−^) helped us to confirm the existence of MPI8•H_2_O.

Several mechanisms on multiple peaks in RP chromatography from compounds with structural characteristics similar to those of MPI8 have been reported. For example, for compounds such as lisinopril, enalapril, and captopril, which are dipeptides containing L-proline, observed multiple chromatographic peaks were attributed to the existence of two rotation isomers with respect to the conformation across the amide bond [34,35,36,37]. Another mechanism of multiple peaks for compounds such as curcumin, an α, β-unsaturated diketone, was confirmed to form keto–enol tautomerization using LC-TOF MS [38]. Third, the formation of a gem-diol was attributed to multiple chromatographic peaks from compounds having a formyl functional group [39,40]. 

A gem-diol, also viewed as the aldehyde (or ketone) hydrates, is an organic compound having two hydroxyl functional groups bound to the same carbon atom. For example, a formaldehyde molecule captured a water molecule and turned into its gem-diol form, also known as methanediol, as the dominant form in an aqueous solution at ambient temperature [41,42,43]. Our TOF MS and MS/MS results revealed that MPI8 formed MPI8•H_2_O in an aqueous matrix and even in a solvent such as acetonitrile, suggesting the formation of an MPI8 gem-diol.

The interactions between water and biological molecules, such as peptides and proteins, are common and often complex [44]. For example, valinomycin is a cyclic peptide, and the formation of valinomycin hydrates via hydrogen bond interactions with carbonyl oxygen was confirmed using scanning tunneling microscopy (STM) with density functional theory (DFT) calculations [45]. Quantum calculations for vibrational states and the energy levels of several peptides and peptide-water complexes were reported. The calculations were for variants of the Amber force field and used the vibrational self-consistent field (VSCF) method, which includes the effects of anharmonicity as well as the interactions between modes. Distinct isomers of a single peptide-H_2_O complex corresponding to different hydrogen bonding sites of the H_2_O were observed [46]. Multiple water molecules could form a complex with peptides, as in the case of dipeptide N-acetyl-L-alanine N-methylamide, which forms a complex with four water molecules [47].

Although we believe that MPI8•H_2_O was more likely a gem-diol rather than a peptide-H_2_O complex, based on the above discussion, the present HR MS/MS spectrum of MPI8•H_2_O did not provide structural information to confirm such a hypothesis. Additional studies are warranted to further characterize the structural conformation of the compound. MPI8•H_2_O was referred to as a water adduct in this study.

### 2.2. Method Development

**Figure 5:**
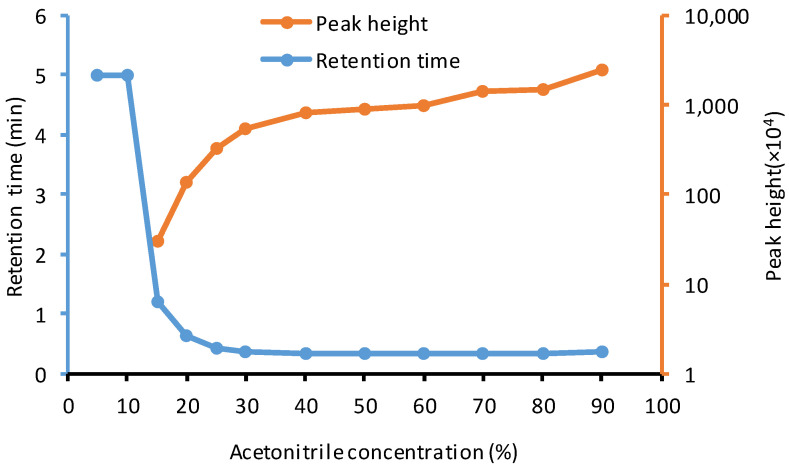
The peak height and retention time of MPI8 (100 ng/mL) on a Kinetex HILIC column in isocratic elution at different concentrations of the mobile phase component of acetonitrile, from 5% to 90%. When the acetonitrile composition in the mobile phase was less than 10%, MPI8 was completely retained on the column, and the retention time mark was 5 min, which was the total running time of the test.

To overcome the problem of multiple peaks, chemical derivatization [37,40] and complicated modifications of chromatographic conditions (mobile phases, temperature, etc.) [34,35,36,39] have been reported. A new strategy—“to co-elute MPI8 and MPI8•H_2_O on a suitable column”—has been proposed here. We searched other types of columns that would have acceptable column retention but not differentiate MPI8•H_2_O from MPI8 in the column separation. We identified a Kinetex hydrophilic interaction liquid chromatography (HILIC) column that achieved this goal of a single good-shaped MPI8 peak with an acceptable retention time for quantification without any interference from MPI8•H_2_O (Figure 2D). Interestingly, MPI8 retention behavior on the Kinetex HILIC column, which is widely considered as a normal phase (NP) column, was similar to that of an RP column. Figure 5 shows the peak height and retention time of MPI8 (100 ng/mL) on a Kinetex HILIC column at different concentrations of the mobile phase component of acetonitrile, from 5% to 90%. When the acetonitrile composition in the mobile phase was less than 10%, MPI8 was completely retained on the column. On the other hand, a mobile phase with >25% acetonitrile would render MPI8 non-retention on the column. Thus, we used an RP chromatographic gradient and the transition *m*/*z* 601.4→157.2 for the quantification of MPI8. The unique behavior of MPI8 on the HILIC column, which resembles a RP characteristic, was most likely due to its hydrophobic nature.

To obtain the optimal signals, the effect of generally used additives in LC-MS/MS analysis, including formic acid, acetic acid, ammonium formate, and ammonium acetate, were tested and compared with non-additive water/acetonitrile as the mobile phases A/B. The water/acetonitrile system showed the highest intensity, and water containing 1 mM ammonium acetate/acetonitrile achieved the closest intensity with better consistency. Therefore, water containing 1 mM ammonium acetate and acetonitrile were chosen as the mobile phases A and B, respectively. 

Due to the rapid conversion of MPI8 sulfonate to MPI8 in vivo, only the active drug MPI8 was detectable following MPI8 sulfonate administration in rats. In addition, there were no identified active metabolites of MPI8. Thus, we only developed an LC-MS/MS assay for MPI8 in rat plasma.

**Figure 6:**
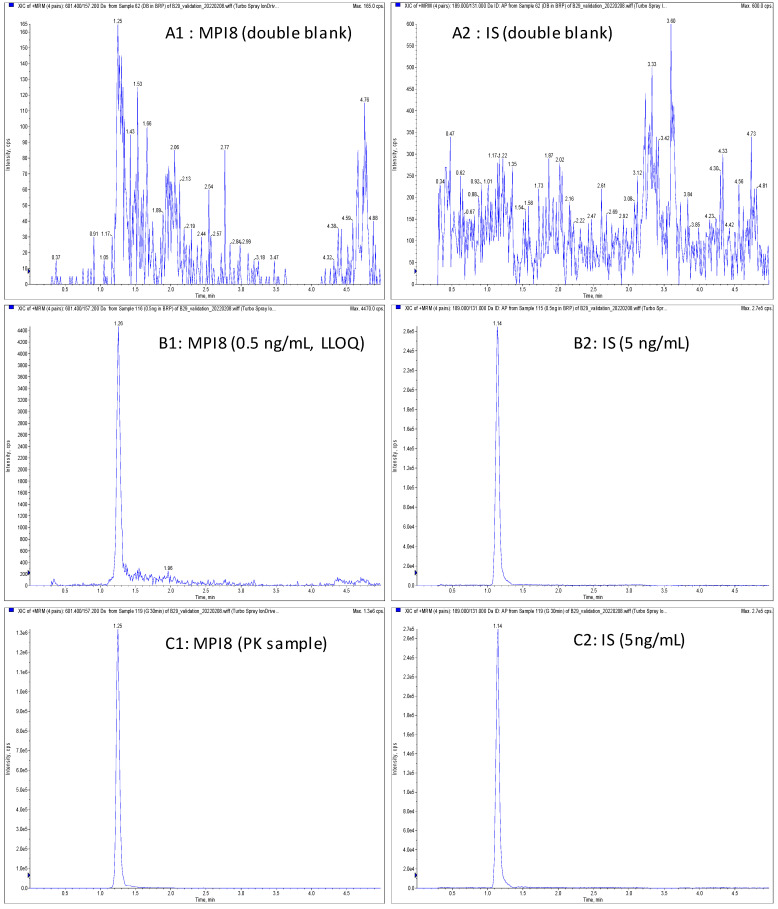
Representative MRM chromatograms: (**A1**) MPI8 in double blank rat plasma, (**A2**) IS in double blank plasma; (**B1**) MPI8 (0.5 ng/mL) spiked in blank rat plasma, (**B2**) IS (5 ng/mL) spiked in blank rat plasma; (**C1**) MPI8 in a rat plasma sample at 30 min after IV administration of MPI8 sulfonate at a single dose of 5 mg/kg, (**C2**) IS (5ng/mL) in a rat plasma sample at 30 min after IV administration of MPI8 sulfonate at a single dose of 5 mg/kg. There was no interference at the retention times of the analyte and IS, and no carryover for both IS (≤5% of average response) and MPI8 (≤20% of LLOQ).

There was no available stable isotope-labeled MPI8. We tested several compounds, including GC376. We selected AP (Figure 1) as an internal standard (IS) based on its appropriate retention time and no interference at the retention times (Figure 6).

### 2.3. Method Validation

The method specificity was assured by analyzing six individual blank plasma samples, and there was no interference at the retention times of the analyte and IS. The selectivity was confirmed by analyzing six individual blank plasma samples at the concentration of the lower limit of quantitation (LLOQ). The back-calculated values for these samples were within ±20.0% of their theoretical value. Chromatograms of a blank rat plasma sample, a blank rat plasma sample spiked with MPI8, and a rat plasma sample taken at 30 min after intravenous administration of MPI8 sulfonate are shown in Figure 6. There was no carryover for both IS (≤5% of average response) and MPI8 (≤20% of LLOQ).

The linearity of the calibration curves was found to be in the range of 0.5–500 ng/mL. A typical calibration curve is presented in Figure S2. The linear regression coefficient of determination (r^2^) were greater than 0.99 in all validation runs.

The accuracy and precision were determined by replicate analyses (*n* = 6) of quality control (QC: 0.5, 1.5, 25 and 400 ng/mL) samples on different validation days. Table 2 summarizes the precision and accuracy results. The intra-day accuracy, expressed by relative error (RE%), was −12.03% to 7.53%; the precision, expressed by the coefficient of variation (CV%), ranged from 7.42% to 10.10%; the inter-day accuracy ranged from −8.44% to 2.16%; and the inter-day precision was between 10.53% to 13.32%. The precision and accuracy were within the acceptance range, according to the FDA bioanalysis guidance [48]. These data suggest that the method is accurate and precise for the quantification of MPI8 in rat plasma (Table 2).

The matrix effects were investigated using the post-extraction spike method, and ranged from 135.9% to 142.8% (Table 3), suggesting the matrix enhanced the signal response of MPI8. Despite the observed matrix effect, the precision (CV%) of the matrix effect was <6.5%. Thus, we expected negligent effect in the quantification of MPI8. Recoveries were calculated by comparing the peak areas in QC samples to those of post-extracted QC samples. The recoveries ranged from 68.3% to 79.9% (Table 3).

The stability of MPI8 is shown in Table 4. No significant degradation occurred under the experimental conditions. When we developed the method, the bench top stability was tested at room temperature (50 ng/mL, *n* = 4); the accuracy (RE%) was −27.6%, suggesting that MPI8 was not stable at room temperature in rat plasma, and the sample should be stored on ice while processing.

Dilution integrity ensures that the dilution of a specimen with a concentration higher than the upper limit of quantification (ULOQ) could result in an accurate quantification. Six 25-fold HQC samples were diluted 25 times and analyzed. The accuracy was −7.67%, and the precision was 3.11%, suggesting that proper dilution is acceptable for the method.

### 2.4. Pharmacokinetic Study

**Figure 7:**
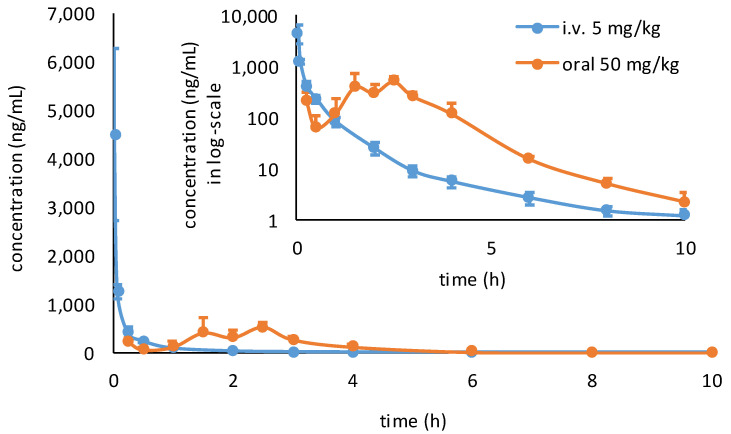
Mean plasma concentration vs. time profiles of MPI8 in rat plasma after i.v. (*n* = 3) and oral (*n* = 3) administration (normal scale plots in normal graph and semi-log plots in inset graph). Twelve time-point plasma samples from rats dosed intravenously and 13 time-point plasma samples from rats dosed orally were collected. Three replicates for each time point were analyzed and reported as mean ± SD for each time-point.

The mean plasma concentration vs. time profiles of MPI8 in adult Sprague-Dawley rats after IV or oral administrations of MPI8 sulfonate are presented in Figure 7. The main pharmacokinetic parameters are listed in Table 5.

Following IV administration, MPI8 had a mean initial plasma concentration of 4482 ng/mL at 2 min after dosing, followed by a tri-exponential decrease. At 10 h post-dose, the MPI8 mean concentration was at 1.24 ng/mL and became undetectable by 24 h. T_1/2_ was 2.75 h.

Following oral administration, MPI8 was quickly absorbed and reached C_max_ of 601 ng/mL in 2.2 h. MPI8 appeared to have a wider window of absorption in the gastrointestinal tract. Two out of three rats showed double peaks around 1.5 and 2.5 h, suggesting that potential drug solubility limited continuous absorption. The mean oral bioavailability of MPI8 was 15.8%.

## 3. Materials and Methods

### 3.1. Chemicals and Reagents

MPI8 was synthesized in-house according to our published methods [14]. Its prodrug MPI8 sulfonate was synthesized by treating MPI8 with sodium bisulfite (see the Supporting Materials). The IS AP and ammonium acetate were purchased from Sigma-Aldrich (St. Louis, MO, USA). LC-MS grade acetonitrile and water were purchased from VWR Chemicals BDH^®^ (Chicago, IL, USA). Blank rat plasma was purchased from BioIVT (Westbury, NY, USA). All other reagents were of analytical reagent grade and were purchased from commercial suppliers.

### 3.2. Identification of MPI8•H_2_O in Rat Plasma

The identification for MPI8•H_2_O was performed on an X500B QTOF mass spectrometer (SCIEX, Framingham, MA, USA) using a Synergi Fusion-RP column (4 µm, 150 × 2 mm, 80 Å, Phenomenex, Torrance, CA, USA). The elution solvents consisted of water (A) and acetonitrile (B). The flow rate was set at 0.4 mL/min. The autosampler and oven temperature were set at 10 °C and 30 °C, respectively. The running time was 10 min. The time program of the gradient was as follows: Phase B was initially at 20% for 0.5 min, increased from 20% to 90% in 4.5 min, kept at 90% for 2 min, decreased to initial concentration (20%) in 1 min, and equilibrated for 2 min. The QTOF MS/MS was equipped with a TurboV ESI ion source, operated in the positive and negative ion modes. TOF-MS and TOF-MS/MS data were acquired using the Information-dependent-acquisition (IDA) mode. IDA QTOF MS, a survey scan performed to collect the information on the precursor ions, was followed by multiple dependent QTOF MS/MS scans for several of the most abundant precursor/candidate ions, which is more suitable to identify potential MPI8 adducts in the matrix. The temperature and spray voltage were set at 500 °C, and 5000 v, respectively. The curtain gas and collision gas (CAD) were set at 30 and 10 psi, and nebulizer gas (gas 1) and heater gas (gas 2) were set at 55 and 60 psi, respectively. The QTOF-MS was set over a *m*/*z* range from 100 to 1000 with an accumulation time of 0.2 s. Declustering potential (DP) and collision energy (CE) were (−)50 v and (−)10 v, respectively. QTOF-MS/MS was set over a *m*/*z* range from 50 to 1000 with an accumulation time of 0.05 s, and DP and CE were (−)50 v and (−)35 v with a 15 v of spread, respectively. Data were acquired by SCIEX OS software 1.6.1.

### 3.3. MPI8 Quantification and Method Validation

#### 3.3.1. Analytical Conditions

The quantification of MPI8 concentrations in extracted plasma samples was performed on a 6500+ Triple Quad LC-MS/MS System using a Kinetex HILIC Column (unbonded silica stationary phase, 2.6 µm, 50 × 2.1 mm, 100 Å, Phenomenex, CA, USA). The optimized method used binary gradient mobile phases with water (containing 1 mM ammonium acetate) as mobile phase A and acetonitrile as mobile phase B. A flow rate of 0.4 mL/min was used with an injection volume of 2 μL. The autosampler and oven temperature were set at 10 °C and 30 °C, respectively. The running time was 5 min. In this study, the HILIC column, widely considered as an NP column, was eluted in a typical RP time program of the gradient: Phase B was initially kept at 5% for 0.2 min, increased from 5% to 90% in the next 1.3 min, then held at 90% for 1 min, and then decreased to 5% in 1.5 min, and kept stably at 5% for 1 min. The unique behavior of MPI8 on a HILIC column, which resembles an RP characteristic, was most likely due to its hydrophobic nature.

Mass spectrometry data were recorded using ESI, positive ion detection, and MRM scanning. The ion spray voltage and temperature were set at 5000 v and 350 °C, respectively. The curtain gas and CAD were set at 45 and 8 psi; gases 1 and 2 were set at 40 and 50 psi, respectively. Data were acquired by Analyst software 1.6.3. The MS/MS parameters for MPI8 and IS are shown in Table 1.

#### 3.3.2. Preparation of Standard and QC Samples

A stock solution of MPI8 (1 mg/mL) was prepared by dissolving the 5 mg of MPI8 in 5 mL of acetonitrile. Working solutions were prepared by diluting the stock solution to 5000, 2500, 500, 250, 50, 15, and 5 ng/mL with acetonitrile. The final effective concentrations for the analyte in constructing standard curves were 500, 250, 50, 25, 5, 1.5, and 0.5 ng/mL. The stock solution was stable for 3 months at −80 °C.

A stock solution of AP (IS) was prepared by dissolving AP in acetonitrile at a 1 mg/mL concentration. The stock solution was stable for 12 months at −80 °C.

The working solutions for QC samples were independently prepared at the concentrations of 5, 15, 250, and 4000 ng/mL, which were ten times that of the effective concentrations: 0.5 (LLOQ), 1.5 (LQC), 25 (MQC), and 400 ng/mL (HQC).

#### 3.3.3. Sample Processing

Eighteen microliters of blank rat plasma, 2 μL of working solution (10×) (or 20 μL of rat plasma samples from pharmacokinetic studies), and 180 μL of acetonitrile (containing 5 ng/mL of IS) were vortex-mixed for 2 min and then centrifuged at 14,000 rpm for 20 min at 4 °C. Eighty μL of the supernatant were transferred to a sample vial for LC-MS/MS analysis. All samples were kept on ice during sample processing.

#### 3.3.4. Method Validation

The analytical method was validated according to the US FDA Bioanalytical Method Validation Guidelines for Industry [48].

Selectivity was assessed by comparing the chromatograms of six different batches of blank plasma with the corresponding spiked plasma.

A standard curve in the form of y = Ax + B was determined by plotting the peak area ratio of MPI8 to IS against known standard concentrations of MPI8. The slope, intercept, and coefficient of determination were estimated using the least-squares linear regression method with a weighting of 1/x.

The triplicate injections of blank samples were conducted following six consecutive injections of ULOQ (500 ng/mL) samples. The peak area of the analyte in the blank sample should be ≤20% of that for the LLOQ samples; the peak area of IS should be ≤5% of that of IS throughout the runs.

The intra-day accuracy and precision were determined by replicate analyses (*n* = 6) of QC samples at four levels: 0.5 (LLOQ), 1.5 (LQC), 25 (MQC), and 400 ng/mL (HQC) on one validation day. Inter-day accuracy and precision were determined by six replicates of QC samples on three consecutive days. The RE% was used to estimate the accuracy and CV% was used to estimate the precision. The calculated intra-day and inter-day accuracy and precision results should be ≤15% for QC levels, except for LLOQ ≤ 20%, to meet the FDA’s bioanalysis validation guidance.

The matrix effect and extraction recovery were determined at three levels (LQC, MQC, and HQC) in four replicates. Matrix effects were evaluated by comparing the peak area of the post-extracted QC samples (by spiking analytes in the extracted analyte-free blank plasma samples) to the peak area of the QC samples in the neat solution. Recoveries were calculated by comparing the mean peak area of spiked QC samples to those of post-extracted QC samples.

The stability of MPI8 was examined by keeping four replicates of the QC samples at room temperature and on ice for 4 h, in the auto-sampler tray at 10 °C for 24 h, and in a freezer at −80 °C for 3 weeks; the freeze-thaw stability was obtained over three freeze-thaw cycles, by thawing at 4 °C for 2–3 h and then refreezing at −80 °C for 12–24 h. The concentration of MPI8 after each storage period was determined using the calibration curve analyzed on the same day. The accuracy was expressed by RE%, and the precision was CV%.

The dilution integrity experiment was required if the sample concentration was higher than the ULOQ. Dilution integrity was confirmed by measuring the accuracy and precision of six replicates of 25-fold HQC samples (10,000 ng/mL) with a 25-fold dilution.

### 3.4. Animal Pharmacokinetic Study

The validated assay was applied in a crossover pharmacokinetic study to evaluate the oral bioavailability of MPI8 sulfonate in rats. The animal study protocol (#9132) was reviewed and approved by the Institutional Animal Care and Use Committee (IACUC) at Texas Southern University on 11 July 2020.

Male adult Sprague-Dawley rats were purchased from Envigo RMS (Indianapolis, IN, USA). Rats were cannulated via jugular vein a day before dosing. Three rats were intravenously administered a single dose of 5 mg/kg MPI8 sulfonate (5 mg/mL in 5% dextrose) via the jugular vein. Serial blood samples (about 0.1 mL) were collected from the cannulated jugular vein into heparin-coating tubes before dosing and at 2 min, 5 min, 15 min, 0.5 h, 1 h, 2 h, 4 h, 6 h, 8 h, 10 h, and 24 h time points after administration. After two weeks of washout period, the three rats were fasted overnight and orally administered a single dose of 50 mg/kg MPI8 sulfonate (20 mg/mL in water). Blood samples were collected before dosing and at 15 min, 0.5 h, 1 h, 1.5 h, 2 h, 2.5 h, 3 h, 4 h, 6 h, 8 h, 10 h, and 24 h time points after administration. Briefly, 12 time-point plasma samples from rats dosed intravenously and 13 time-point plasma samples from rats dosed orally were collected. Plasma samples were separated immediately by centrifugation of the blood samples at 13,000 rpm for 3 min and kept at −80 °C until analysis. The samples were processed following the procedure in Section 3.3.3. Three replicates for each time point were analyzed and the results were reported as mean values with standard deviation (SD) for each time point.

The pharmacokinetic parameters for each rat were estimated using Phoenix WinNonlin v8.3 software (Pharsight Corporation, Mountain View, CA, USA). Non-compartmental analysis was used to determine the pharmacokinetic parameters of MPI8. The pharmacokinetic parameters, including the area under the plasma concentration–time curve during the period of measurable observation (AUC_0−10h_), the area under the plasma concentration–time curve extrapolated to infinity (AUC_0−inf_), the apparent volume of distribution (Vz/F), the apparent clearance (CL/F), the total body mean residence time (MRT), and the terminal elimination half-life (T_1/2_) were estimated. The oral bioavailability (F) was calculated according to the following equation:

(1) $$F=\frac{AUC_{oral}\times Dose_{i.v.}}{AUC_{i.v.}\times Dose_{oral}}$$

## 4. Conclusions

MPI8, a potent tri-peptidyl aldehyde-based anti-SARS-CoV-2 agent, forms a water adduct MPI8•H_2_O in aqueous and biological media. MPI8•H_2_O was identified using an accurate mass spectrometer. On common RP columns, MPI8•H_2_O interfered significantly with LC-MS/MS quantification of MPI8 in rat plasma. A HILIC column was identified as an optimal column for LC-MS quantification of MPI8 in rat plasma. Our study would be useful as a reference for developing LC-MS/MS assays for similar small peptidyl aldehyde-based compounds.

## Figures and Tables

**Table 1:** Compound dependent parameters for MPI8 and AP.

	Q1 (*m*/*z*) (Da)	Q3 (*m*/*z*) (Da)	DP (v)	EP (v)	CE (v)	CXP (v)
MPI8	601.4	157.2 *	60	5	30	5
		545.3	60	5	20	5
AP (IS)	189	131	70	5	28	5

Q1: parent ion; Q3: product ion; DP: declustering potential; EP: entrance potential; CE: collision energy; CXP: collision cell exit potential; *: used for quantification.

**Table 2:** Intra-day and inter-day accuracy and precision of MPI8 in rat plasma.

Nominal Concentration (ng/mL)	Intra-Day (*n* = 6)			Inter-Day (*n* = 18)		
	Observed Concentration (Mean ± SD)	Accuracy (RE%)	Precision (CV%)	Observed Concentration (Mean ± SD)	Accuracy (RE%)	Precision (CV%)
0.5 (LLOQ)	0.44 ± 0.04	−12.03	10.10	0.46 ± 0.06	−8.44	12.03
1.5 (LQC)	1.48 ± 0.15	−1.44	9.92	1.51 ± 0.16	0.93	10.53
25 (MQC)	26.88 ± 2.07	7.53	7.69	25.54 ± 3.07	2.16	12.01
400 (HQC)	378.17 ± 28.05	−5.46	7.42	393.61 ± 52.43	−1.60	13.32

**Table 3:** Recovery and matrix effect of MPI8 and AP.

	Nominal Concentration (ng/mL)	Matrix Effect (%) (*n* = 4)	Recovery (%) (*n* = 4)
MPI8	1.5	135.92 ± 8.87	79.88 ± 5.66
	25	138.43 ± 8.45	70.92 ± 3.76
	400	142.78 ± 3.48	68.22 ± 2.22
AP (IS)	5	94.54 ± 6.55	97.62 ± 6.12

**Table 4:** Stability data for MPI8 in rat plasma.

Stability Test	Nominal Concentration (ng/mL)	Calculated Concentration (ng/mL) (*n* = 6)		
		Mean ± SD	(RE%)	(CV%)
Auto-sampler (10 °C 24 h)	1.5	1.44 ± 0.11	−4.33	7.81
	25	26.8 ± 1.91	7.20	7.14
	400	458.5 ± 47.18	14.63	10.29
Bench top (4 h, 4 °C)	1.5	1.35 ± 0.11	−9.87	8.18
	25	21.3 ± 0.12	−14.80	0.57
	400	340.25 ± 14.03	−14.94	4.12
Freeze and thaw (−80 °C to 4 °C, 3 cycles)	1.5	1.33 ± 0.08	−11.17	6.05
	25	23.5 ± 1.41	−6.00	6.02
	400	362.2 ± 11.56	−9.45	3.19
Long-term (−80 °C, 20 days)	1.5	1.44 ± 0.19	−4.22	12.96
	25	25.18 ± 1.37	5.43	0.73
	400	399 ± 17.61	−0.25	4.41

**Table 5:** Pharmacokinetic parameters of MPI8 after IV and oral administration of MPI8 sulfonate to rats (data derived from NCA).

Parameters	Mean ± SD	
	i.v. (5 mg/kg) (*n* = 3)	Oral (50 mg/kg) (*n* = 3)
C_max_	-	600.67 ± 75.04
T_max_	-	2.17 ± 0.58
AUC_0−10 h_ (h∙ng/mL)	613.06 ± 15.40	1177.26 ± 290.10
AUC_0−inf_ (h∙ng/mL)	617.84 ± 15.68	1181.62 ± 287.53
T_1/2_ (h)	2.75 ± 0.53	1.22 ± 0.46
CL/F (mL/h/kg)	6606.93 ± 213.82	43,984.84 ± 10,389.13
Vz/F (ml/kg)	26,324.66 ± 5882.81	81,570.63 ± 48,751.79
MRT (h)	0.54 ± 0.10	2.52 ± 0.16
F (%)	-	15.76 ± 4.35

C_max_: maximum drug concentration; T_max_: time to reach maximum drug concentration; AUC_0−10h_: the area under the plasma concentration–time curve during the period of measurable observation; AUC_0−inf_: the area under the plasma concentration–time curve extrapolated to infinity; T_1/2_: terminal elimination half-life; CL/F: apparent clearance; Vz/F: the apparent volume of distribution during the terminal phase; MRT: mean residence time; F: oral bioavailability; -: not applicable.

## Data Availability

The data have been presented in the main text and in the Supplementary Materials.

## Supplementary Materials

The following supporting information can be downloaded at: https://www.mdpi.com/article/10.3390/ph15060676/s1, Figure S1: ^1^H NMR (400 MHz, Methanol-d_4_) spectra of MPI8 sulfonate. Figure S2: A typical calibration curve of MPI8 (the linear range 0.5–500 ng/mL, r = 0.9999).
