# Supplementary material for: Accurate Mass Identification of an Interfering Water Adduct and Strategies in Development and Validation of an LC-MS/MS Method for Quantification of MPI8, a Potent SARS-CoV-2 Main Protease Inhibitor, in Rat Plasma in Pharmacokinetic Studies
Source: Pharmaceuticals (Basel). 2022 May 27;15(6):676. doi: 10.3390/ph15060676 (PMC9228185; doi:10.3390/ph15060676)
Supplement: Supplementary file 1 [file pharmaceuticals-15-00676-s001.zip › pharmaceuticals-1722470-supplementary.pdf]

Supplementary Material

Accurate Mass Identification of an Interfering Water Adduct and Strategies in Development and Validation of an LC-MS/MS Method for Quantification of MPI8, a Potent SARS-CoV-2 Main Protease Inhibitor, in Rat Plasma in Pharmacokinetic Studies

Yang Wang [1], Huan Xie [1], Yugendar R. Alugubelli [2], Yuying Ma [2], Shiqing Xu [2], Jing Ma [1], Wenshe Liu [2,*] and Dong Liang [1,*]

[1] Department of Pharmaceutical Sciences, College of Pharmacy and Health Sciences, Texas Southern University, Houston, TX, 77004, USA; yang.wang@tsu.edu (Y.W.); huan.xie@tsu.edu (H.X.); jing.ma@tsu.edu (J.M.); dong.liang@tsu.edu (D.L.)

[2] Department of Chemistry, Texas A&M University, College Station, TX 77843, USA; namaswi@tamu.edu (Y.R.A.); yuyingma.cn@gmail.com (Y.M.); shiqing.xu@tamu.edu (S.X.); wsliu2007@tamu.edu (W.L.)

* Correspondence: dong.liang@tsu.edu (D.L.); wsliu2007@tamu.edu (W.L.); Tel.: +1-713-313-1885 (D.L.); +1-979-845-1746 (W.L.)

The supplementary document contains the following information regarding the studies described in the manuscript:

S1: Synthetic procedures of MPI8-Sulfonate

S2: A typical standard curve of MPI8 in rat plasma

S1. Synthesis of MPI8-Sulfonate

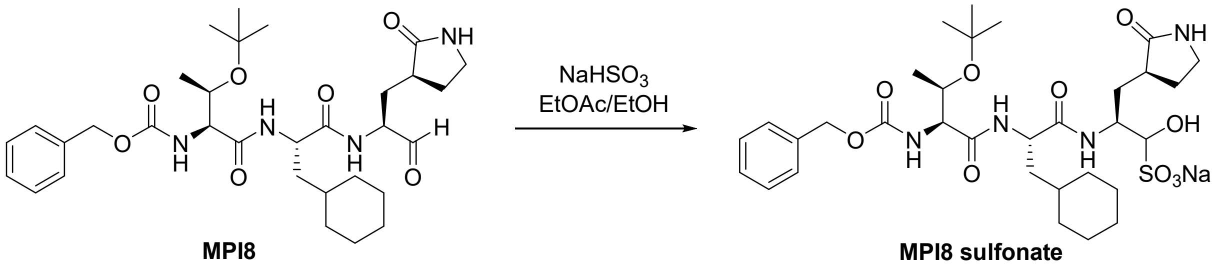

MPI8 **MPI8 sulfonate**

To a solution of MPI8 (100 mg, 0.17 mmol, 1.0 equiv) in dry ethyl acetate (5 mL) was added absolute ethanol (5 mL) with stirring, followed by a solution of sodium bisulfite (53 mg, 0.51 mmol, 3.0 equiv) in water (2 mL). The reaction mixture was stirred for 3 h at 50 °C. The reaction mixture was allowed to cool to room temperature and then vacuum filtered. The solid was thoroughly washed with absolute ethanol, and the filtrate was dried over anhydrous sodium sulfate, filtered, and concentrated to yield yellowish oil. The oily product was treated with ethyl ether to form white solid (95 mg, 80%). ^{1}H NMR (400 MHz, Methanol-d_4) δ 7.52 – 7.20 (m, 5H), 5.23 – 5.08 (m, 2H), 4.52 – 4.39 (m, 2H), 4.16 – 4.07 (m, 2H), 4.01 – 3.90 (m, 1H), 3.32 – 3.23 (m, 2H), 2.48 – 2.38 (m, 1H), 2.37 – 2.26 (m, 1H), 2.08 – 1.96 (m, 1H), 1.87 – 1.50 (m, 9H), 1.49 – 1.27 (m, 4H), 1.23 (s, 9H), 1.18 – 1.13 (m, 3H), 1.06 – 0.89 (m, 2H).

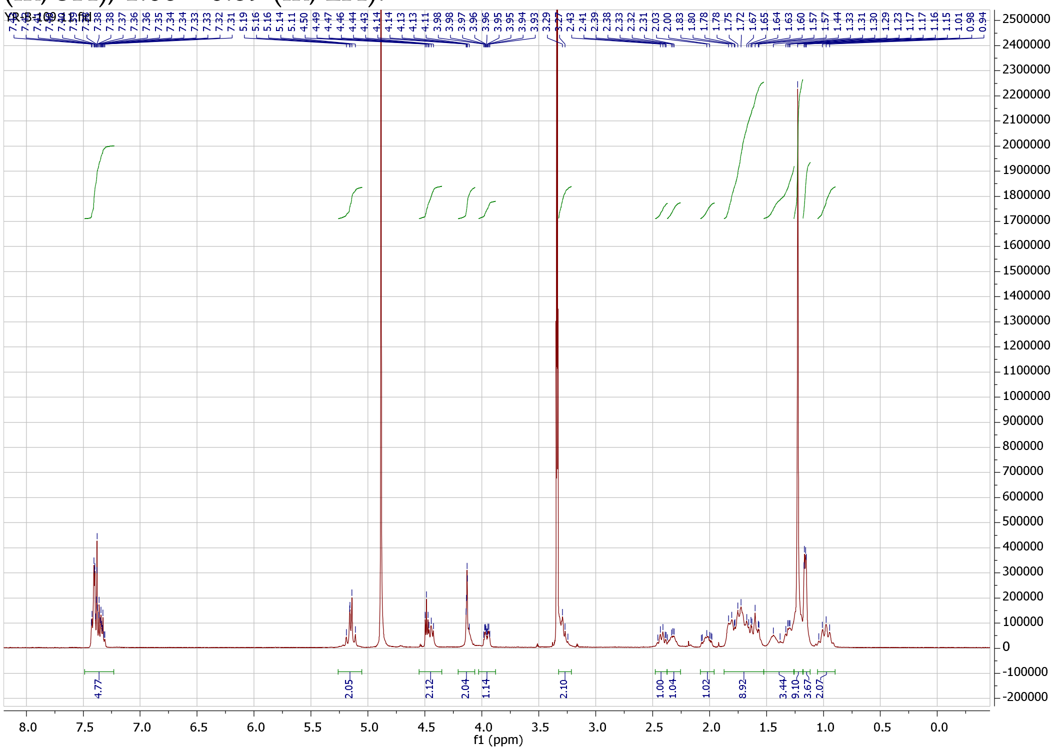

Figure S1. ^{1}H NMR (400 MHz, Methanol-d_4) spectra of MPI8 sulfonate.

S2. A Typical Calibration Curve

The linearity of the calibration curves was found in the range of 0.5 – 500 ng/mL. A typical calibration curve of MPI8 was presented in Figure S2. The linear regression correlation coefficients were greater than 0.99 in all validation runs.

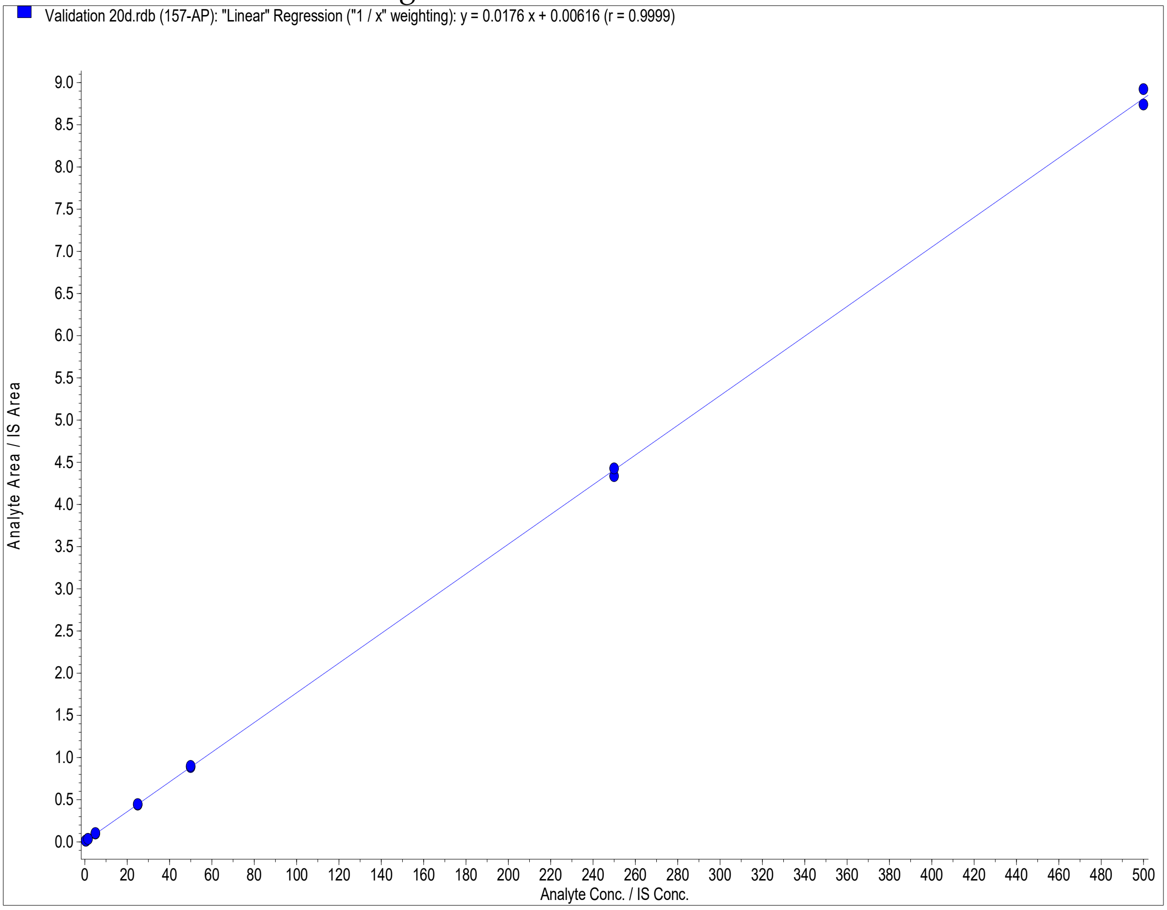

Figure S2. A typical calibration curve of MPI8 (the linear range 0.5-500 ng/mL, r=0.9999). The transition m/z 601.4→157.2 was used for the quantification. The linear regression correlation coefficients were greater than 0.99 in all validation runs.
